# Mesodermal mesenchymal polyp; wide introitus in young female^☆^

**DOI:** 10.1016/j.ijscr.2024.110084

**Published:** 2024-07-31

**Authors:** Khalid Akkour, Dana Aldabeeb, Metab Alkubeyyer, Mohammed Alswayyed, Ghadeer Alshaikh

**Affiliations:** a Department of Obstetrics and Gynecology, College of Medicine, King Saud University, Riyadh, Saudi Arabia; b Department of Pathology, College of Medicine, King Saud University, Riyadh, Saudi Arabia; c Department of Radiology, College of Medicine, King Saud University, Riyadh, Saudi Arabia

**Keywords:** Mesodermal mesenchymal polyp, Fibroepithelial stromal polyps, Wide introitus

## Abstract

**Introduction and importance:**

Mesodermal mesenchymal polyps (Fibroepithelial stromal polyps) are mesenchymal embryological structures that remain and grow to be an apparent polyp-like lesion in females of reproductive age. We present this case of mesodermal mesenchymal polyp in a young female arising at the hymenal ring of the vagina making the introitus very wide. We highlighted in our case the importance of recognizing mesenchymal lesions and their differential diagnosis to provide the patients with optimal care.

**Case presentation:**

A 24-years-old single female presented with a painless vaginal mass since birth, that increased in size after puberty. Upon examination, she was found to have an irregular smooth mass with around 4 × 4 cm of it protruding outside the vagina and easily reducible disfiguring and making the introitus wide. After performing Magnetic resonance imaging (MRI), findings were Suggestive of a Vulvovaginal mesenchymal tumor likely aggressive angiomyxoma. The patient underwent surgical vaginal mass excision, with hymenal repair, posterior and anterior vaginal wall reconstruction. The final diagnosis confirmed by tissue pathology was mesodermal mesenchymal polyp.

**Clinical discussion:**

Fibroepithelial polyps of the vagina (FEPV) is a rare benign neoplasm and most commonly asymptomatic apart from painless mass protruding or disfiguring the sensitive area. The variety of mesenchymal lesions that occur at the vulvovaginal region can be very challenging histopathologically and surgically due to their rarity and lack literature.

**Conclusion:**

Fibroepithelial polyps of the vagina (FEPV) remain an infrequent entity of pathologies affecting the female urogenital tract. We reported a rare case of concomitant FEPV and wide introitus affecting a young woman physically and psychologically. Therefore, preoperative clinical assessment and surgical approach along with psychological support is critical to provide the patient with the best outcome.

## Highlights

• The variety of mesenchymal lesions that occur at the vulvovaginal region can be very challenging histopathological and surgically due to their rarity and lack literature.
• We present this case of meosdermal mesenchymal polyp in a young female arising at the hymenal ring of the vagina making the introitus very wide.
• Managing patient with Mesodermal mesenchymal polyp pre operatively and Surgical approach.
• We highlighted in our case the importance of recognizing mesenchymal lesions and their differential diagnosis to provide the patients with optimal care.

## 1 Introduction

The diversity of mesenchymal lesions that occur at the vulvovaginal region can be very challenging histopathologically and surgically due to their rarity and deficient literature. A mesodermal mesenchymal polyp (Fibroepithelial stromal polyps) is a mesenchymal embryological structure that remains and grows to be an apparent polyp like lesion in females of reproductive age. The vagina is the most common location. However, it may be found at the vulva, cervix and less commonly in extra-genital sites. These polyps are hormone-dependent and are mostly diagnosed during pregnancy. However, it can be seen in pre or postmenopausal women on hormonal replacement therapy [1]. These lesions can be polypoidal or pedunculated and usually present as a single tumor rather than multifocal lesions. Presenting symptoms varies, including pressure-like feeling or discomfort, bleeding, or discharge. Based on their size they can be further divided to either skin tags if they are millimetric, polyps where they are slightly bigger but less than 5 cm, or giant lesions when the size exceeds 5 cm [2]. Until writing this report, there are only a few reported cases of giant fibroepithelial stromal polyps of the vulva [[3], [4], [5]]. Here, we present a case of mesodermal mesenchymal polyp in a young female arising at the hymenal ring of the vagina making the introitus very wide and disfigured. This case report has been reported in line with the SCARE criteria [6]. To our knowledge, this is the first case report representing wide introitus in young females, even though she had no prior sexual intercourse. She has presented after seeing multiple physicians who misdiagnosed her as having a collagen-deficient disorder, thus having step-by-step approach is essential.

## 2 Case presentation

**Fig. 1:**
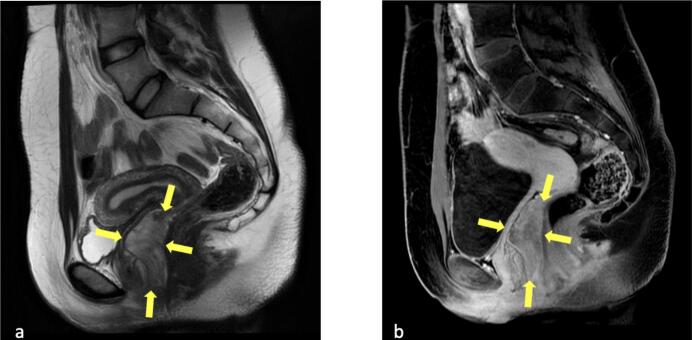
Sagittal T2WI of pelvis showing posterior vaginal mass with laminated appearance of moderate to high signal in T2WI (Yellow arrows in a) corresponding to mild enhancement in post IV contrast T1WI (b).

**Fig. 2:**
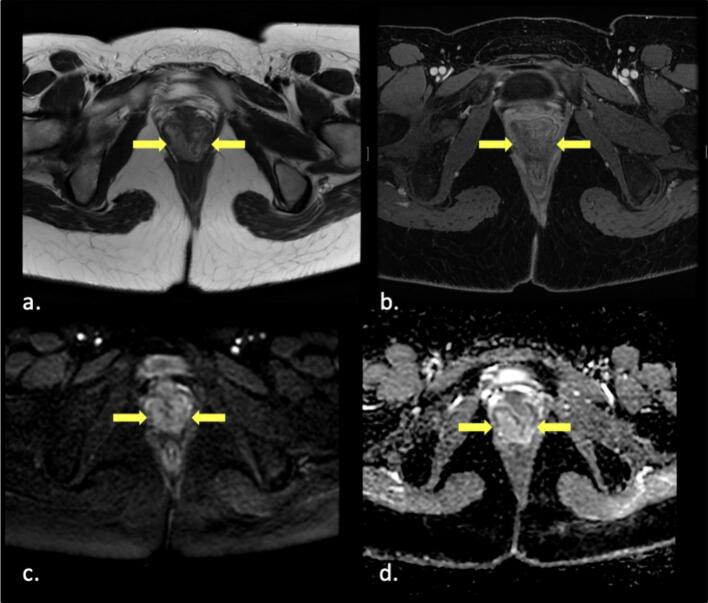
(a.) Axial T2WI at the level of distal vagina showing laminated appearance of posterior vaginal mass displacing the collapsed vaginal lumen anteriorly. (b.) corresponding T1WI post IV contrast showing minimal enhancement. (c and d) corresponding DWI showing no diffusion restriction.

A twenty-four-year-old single female with no history of sexual relations, she had her menarche at the age of fourteen with regular menstrual cycle. She was born full term and her mother reported an uneventful pregnancy course with no history of use of any medication or herbal use during pregnancy. She is not known to have any medical illnesses and her surgical history was significant for laparoscopic ovarian cystectomy four years before her presentation. She presented to the urogynecology clinic complaining of a painless vaginal mass. She reported that this mass has been present since birth, and was very small, but after puberty it started gradually to grow and protrude from her vagina, she never had vaginal pain or discharge. Upon examination, she was found to have an irregular smooth mass with around 4 × 4 cm of it protruding outside the vagina and easily reducible, the root of the mass seemed to be inside the vagina but was not determined as the patient started to have pain and discomfort while examining her. There were no ulcerations or fungations. She was admitted for workup and a Magnetic resonance imaging (MRI) of the pelvis was requested and showed a laminated appearance predominantly hyperintense in T2WI with low signal thin streaks extraluminal vaginal mass showing subtle enhancement post IV contrast administration without fatty component. It is seen along the posterior aspect of the vagina measuring 8x3cm, extended from the introitus up to the level of the anterior vaginal fornix pushing the vagina anteriorly with no evidence of invasion of the anal canal or adjacent muscles. Both ovaries and uterus are unremarkable. The urinary bladder and rectum are unremarkable. No pelvic free fluid. No size significant lymphadenopathy. No aggressive bone lesions. The findings are suggesting a Vulvovaginal mesenchymal tumor likely aggressive angiomyxoma (Fig. 1, Fig. 2).

**Fig. 3:**
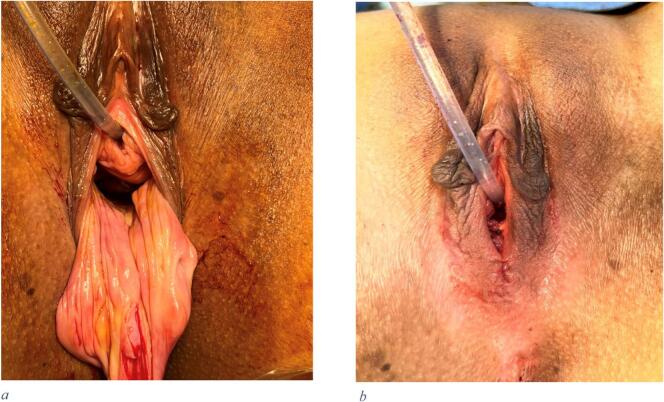
(a.) A disfiguring mass 5x5x5 cm posteriorly and 2 × 2 cm anteriorly involving the hymen and the distal third of urethra making the introitus very wide although sexually not active. (b.) post vaginal mass excision, with hymenal repair, posterior and anterior vaginal wall reconstruction.

The patient was counselled, consented, and taken the same week for examination under anaesthesia which revealed the exact description mentioned in the MRI. A disfiguring mass 5 × 5 × 5 cm posteriorly and 2 × 2 cm anteriorly involving the hymen and the distal third of the urethra making the introitus very wide although sexually not active (Fig. 3).

She was consented in addition to the examination under general anaesthesia, for surgical resection and reconstruction. The patient was brought into the theatre and placed in a lithotomy position, identification of the mass borders using Alice forceps was done, and careful inspection and palpation of the mass before dissection of the mass from the vaginal epithelium. Post vaginal mass excision, with hymenal repair, posterior and anterior vaginal wall reconstruction was done (Fig. 3).

**Fig. 4:**
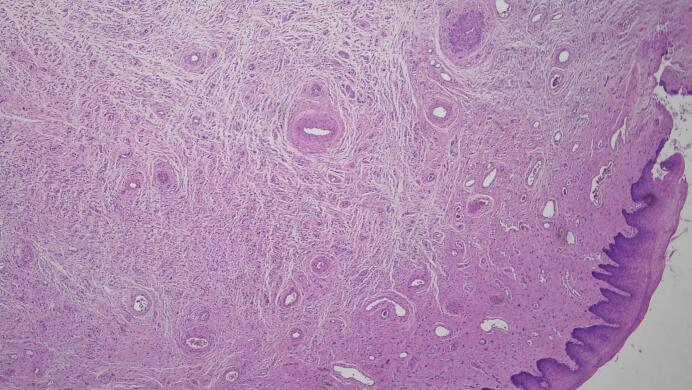
H&E, 20× Hematoxylin and eosin-stained tissue shows bland hypocellular spindle cell proliferation includes fibroblast and smooth muscle in background of thick wall blood vessels and prominent nerves bundles.

Histopathological examination showed a benign polypoid lesion consistent with mesodermal mesenchymal polyp with no evidence of malignancy. Further sectioning shows polypoidal lesion composed of bland hypocellular spindle cell proliferation which includes fibroblast and smooth muscle in background of thick wall blood vessels and prominent nerve bundles. By immunohistochemistry, the smooth muscle cells are positive for Desmin and SMA. Fibroblastic cells are positive for CD34, ER and PR. CD34 highlighted blood vessels and S100 protein highlighted the nerve bundles. The lesional cells are negative for cytokeratin. The morphologic and immunophenotypic findings are most consistent with mesodermal mesenchymal polyp (Fig. 4).

## 3 Discussion

Benign and malignant Neoplasms arising from the vagina are unusual entities. The presenting symptoms of vaginal neoplasms differ according to the nature of the tumor, and patients may present with pressure feeling, dyspareunia, tissue protruding from the vulva, pain, and per vaginal bleeding/discharge [7]. Fibroepithelial polyps of the vagina (FEPV) is a rare benign neoplasm and most commonly asymptomatic apart from painless mass protruding or disfiguring the sensitive area. It most commonly originates from vulvovaginal subepithelial mesenchymal cells hence comes the other name described in the literature (mesodermal menchymal polyp). The most common specific location reported is the Vagina followed by the cervix and the vulva [4,8,9]. Extra vulvovaginal lesions are rare, it has been found in the breast which is considered unusual presentation of FEPV [10,11]. The pathogenesis of FEPV is unknown. Yet, it has been associated with hormonal stimulation, as it most commonly occurs during pregnancy and subsides or shrinks after giving birth. Among the cases reported of FEPV, it was found that these lesions are affected by the hormonal status of the patient as reproductive age or patients consuming hormones are counting for the majority of cases [12]. FEPV usually arise in the lower genital tract of females in most of cases [4,13]. Almost all reported cases were for adult patients, one of the cases was of valvular Fibroepithelial stromal polyp in a 7-month-old girl [14]. Our patient was born with the mass however it was small and discovered incidentally by her pediatrician at birth; she reported that the size has substantially increased after puberty which further supports the hormonal-dependent nature of such tumors. Moreover, Hartmann CA et al. supported this hormonal theory by performing immunohistochemistry reporting the expression of vimentin, desmin, estrogen and progesterone receptors in vaginal fibroepithelial stromal polyps where he found most of these lesions expressed desmin and steroid hormone receptors but no muscle-specific actin or macrophage markers [15]. Microscopically, fibroepithelial stromal polyp have been described to have a fibrovascular core, stroma with pedunculated or polypoid proliferation and overlayed by squamous epithelium. Stromal cells have been described to be spindle or stellate-shaped and may include multinucleated cells. In general, it has been described as bland nuclear features. Moreover, stromal cells tend to aggregate in the stromal-epithelial junction and surround blood vessels of the central fibrovascular core. Hyperplasia of the squamous epithelium laying on the stromal proliferation is a common finding with positive staining to desmin, estrogen and progesterone receptors, and occasionally to smooth muscle actins [13,16]. FEPV is considered a benign pathology and has not been reported to have destructive or metastasis features. In our case, FEPV in a young female who has no previous sexual intercourse caused a widening in genital hiatus and wide introitus, which is not a common presentation. Tissue trauma, and vaginal stretching caused by the mass effect on the vagina, could play a role in the patient presentation. After a carful history and preoperative assessment, including MRI findings, FEPV was treated by local excision and meticulous cosmetic repair. The surgery outcome was successful and resulted in a complete relief of patient symptoms including the psychological effect this mass was reflecting on the patient as she was losing hope in getting sexually active and entering a relationship in her future. The post-operative review should include a full history review and a clinical examination including a bimanual exam and rectovaginal examination for the recurrence of the mass. She was seen at one, 6 and 12 months postoperatively as an outpatient where she showed an amazing recovery from both surgical and psychological points of view.

## 4 Conclusion

Mesodermal mesenchymal polyp (FEPV) remains an infrequent entity of pathologies affecting the female urogenital tract. We reported a rare case of concomitant FEPV and wide introitus affecting a young woman physically and psychologically. We believe that no panic approach followed by proper work up and careful preoperative planning and intraoperative meticulous technique will help these patients recover with the least possible physical damage and the highest levels of self-confidence and satisfaction. Similar cases should be always managed in centers where expertise in both histopathology and gynecology is available as that plays central and crucial role in managing such cases.

## Ethical approval

Ethical approval is not required for case reports in King Saud University, Riyadh, Saudi Arabia.

## Funding

None.

## Author contribution

Khalid Akkour: reviewing the manuscript

Dana Aldabeeb: writing the manuscript

Muteb Alkubeyyer: reviewing the pathology

Mohammed Alsawayyed: reviewing the manuscript

Ghadeer Alshaikh, reviewing the manuscript

## Guarantor

Dr. Dana Aldabeeb

## Consent

Written informed consent was obtained from the patient for publication and any accompanying images. A copy of the written consent is available for review by the Editor-in-Chief of this journal on request.

## Conflict of interest statement

None.
